# Prediction of Rapid Chloride Penetration Resistance to Assess the Influence of Affecting Variables on Metakaolin-Based Concrete Using Gene Expression Programming

**DOI:** 10.3390/ma15196959

**Published:** 2022-10-07

**Authors:** Muhammad Nasir Amin, Muhammad Raheel, Mudassir Iqbal, Kaffayatullah Khan, Muhammad Ghulam Qadir, Fazal E. Jalal, Anas Abdulalim Alabdullah, Ali Ajwad, Majdi Adel Al-Faiad, Abdullah Mohammad Abu-Arab

**Affiliations:** 1 Department of Civil and Environmental Engineering, College of Engineering, King Faisal University, Al-Hofuf 31982, Saudi Arabia; 2 Department of Civil Engineering, University of Engineering and Technology, Mardan 23200, Pakistan; 3 Department of Civil Engineering, University of Engineering and Technology, Peshawar 25120, Pakistan; 4 Department of Environmental Sciences, Abbottabad Campus, COMSATS University Islamabad, Abbottabad 22060, Pakistan; 5 Department of Civil Engineering, Shanghai Jiao Tong University, Shanghai 200240, China; 6 Civil Engineering Department, University of Management and Technology, Lahore 54770, Pakistan; 7 Department of Chemical Engineering, College of Engineering, King Faisal University, Al-Ahsa 31982, Saudi Arabia

**Keywords:** rapid chloride penetration resistance, metakaolin, gene expression programming, compressive strength of concrete, sensitivity, parametric analysis

## Abstract

The useful life of a concrete structure is highly dependent upon its durability, which enables it to withstand the harsh environmental conditions. Resistance of a concrete specimen to rapid chloride ion penetration (RCP) is one of the tests to indirectly measure its durability. The central aim of this study was to investigate the influence of different variables, such as, age, amount of binder, fine aggregate, coarse aggregate, water to binder ratio, metakaolin content and the compressive strength of concrete on the RCP resistance using a genetic programming approach. The number of chromosomes (N_c_), genes (N_g_) and, the head size (H_s_) of the gene expression programming (GEP) model were varied to study their influence on the predicted RCP values. The performance of all the GEP models was assessed using a variety of performance indices, i.e., R^2^, RMSE and comparison of regression slopes. The optimal GEP model (Model T3) was obtained when the N_c_ = 100, H_s_ = 8 and N_g_ = 3. This model exhibits an R^2^ of 0.89 and 0.92 in the training and testing phases, respectively. The regression slope analysis revealed that the predicted values are in good agreement with the experimental values, as evident from their higher R^2^ values. Similarly, parametric analysis was also conducted for the best performing Model T3. The analysis showed that the amount of binder, compressive strength and age of the sample enhanced the RCP resistance of the concrete specimens. Among the different input variables, the RCP resistance sharply increased during initial stages of curing (28-d), thus validating the model results.

## 1. Introduction

Concrete is a widely used construction material owing to its high compressive strength, ability to mold in any desired shape, and easy availability of its constituent materials. The different mechanical and durability properties of concrete depend upon its constituent materials, such as; (i) gradation, (ii) physical properties of fine and coarse aggregates, (iii) type of cement, (iv) other pozzolanic materials used in conjunction with cement, and (v) the amount of water added to it, among others [1,2,3]. For instance, cement and water react to form binder gel, such as, calcium-silicate hydrate (C-S-H) and calcium-aluminate hydrate (C-A-H), in addition to formation of portlandite. The binder gel acts as a glue to hold the fine and coarse aggregates together [4,5]. Pozzolanic materials, such as fly ash [6], silica fume [7], blast furnace slag [8], pumice [9], metakaolin (MK) [10], etc., have been used in mortar and concrete since they ameliorate the mechanical and durability properties. This also results in the formation of secondary binder gel (i.e., C-S-H, C-A-H or C-A-S-H) depending upon the chemical composition of the pozzolanic material, thus improving the mechanical and microstructural characteristics of concrete [11,12].

MK is a highly reactive pozzolanic material that has been extensively used for performance improvement of mortar and concrete. It is produced by the calcination of kaolinite at elevated temperature ranging between 700–900 °C [13]. Unlike other pozzolanic materials, such as, fly ash, blast furnace slag and silica fume, which are directly obtained from industrial wastes, MK is produced under a controlled environment. Therefore, myriad studies have been conducted to study the influence of MK on pore size distribution and compressive strength of cementitious composites [14,15,16]. It was found that MK-incorporated concrete attained high early strength by accelerating the hydration process and subsequent reaction with portlandite due to MK’s filler effect [17]. This indirectly leads to improved performance of concrete against harsh environmental conditions. For example, Parande et al. [18] found that the water absorption and corrosion resistance of MK-based concrete specimens were reduced in case of 15% cement replacement. The reduced porosity and dense microstructure of the specimens resist chloride and sulfate attacks efficaciously [19]. This implies that the resistance against chloride significantly depends on the mechanical and microstructural characteristics of concrete. The rapid chloride penetration test (RCPT) is a measure of electric conductivity of a concrete sample, and can be determined in accordance with ASTM C 1202 [20]. During this test, the instrument robustly measures the resistance of a particular specimen to chloride ion ingress, which is an indirect measure of the permeability. The microstructural aspects of concrete specimens may be correlated with the mix design of concrete containing cementitious materials. Badogiannis et al. [21] and Reza et al. [22] concluded that the replacement of cement with MK significantly reduced the chloride ingress. This implies that inclusion of MK alongside other constituent materials of concrete tends to quantitatively correlate with the resistance against chloride penetration.

As stated earlier, the performance of cementitious composites is highly dependent on the constituent materials and their mixing proportions. Hence, several trials must be conducted to assess the influence of the properties and the amount of a particular constituent on the cementitious composites. However, such experiments are laborious, as well as time- and resource-consuming [23]. Recently, artificial intelligence (AI) techniques have gained popularity due to their quick learning abilities to model a particular process or phenomenon [24]. These abilities enable the AI model to accurately predict the output considering a number of inputs [25]. For example, Baykasoglu et al. [26] used an artificial neural network (ANN) and gene expression programming (GEP) for predicting the compressive strength of high-strength concrete. Topcu et al. [27] deployed an ANN and adaptive neuro-fuzzy inference system (ANFIS) to estimate the compressive strength of cement mortar containing MK. Following the same methodology, Saridemir [28] studied the influence of fly ash on the compressive strength of concrete. In addition to these AI models, a variety of other models, such as multi-layer neural network (MLNN) [29,30], extreme learning machine (ELM) [31], decision tree (DT) and gradient boosting tree (GBT) models [32] have been successfully used for modelling the compressive strength of concrete with different constituents. Similarly, Kumar et al. [33] successfully employed a multivariate adaptive regression spline and minimax probability machine regression approach to study the influence of elevated temperature curing, fly ash and silica fumes on the RCPT value of self-compacting concrete. Ge et al. [34] employed hybrid models, i.e., random forest combined with particle swarm optimization, whale optimization algorithm and Harris hawk optimization technique for predicting the RCPT of self-compacting concrete by considering different input parameters such as the amount of cement, fly ash, silica fumes, ratio of coarse to fine aggregate, water-to-cement ratio and temperature. It was observed that the random forest-based Harris hawk optimization hybrid model outperformed other models with the highest value of R^2^ = 0.98 and 0.96, and RMSE = 28.6 and 41.4 for the training and testing phases, respectively. Similarly, Yaman et al. [35] employed ANN for predicting the constituents of self-compacting concrete. For this purpose, two different methodologies viz., ANN model with multi input–multi output and ANN model with multi input–single output network were developed using 28 days of compressive strength and diameter of slump flow as the input parameters. It was found that the ANN model with multi input–single output methodology produced better results for the outputs, as evident from their higher R^2^ values (0.63–1.0).

A literature survey found that different AI models exhibit the capability to model the mechanical properties of concrete containing different constituents; however, there are some problems associated with their prediction capabilities, such as producing unexpected outcomes for newer datasets and overfitting of data, which inhibit their extensive use. Similarly, ANN and a few other traditional machine learning (ML) techniques are considered as black-box models [36,37], since these predict output(s) from the input(s) without revealing information about its internal workings, such as how the predictions were produced, and other information related to the influencing variables. Contrary to this, white box models do not have these shortcomings, and the related information about their workings and the influencing variables can be extracted. For example, GEP is a white-box model whose algorithm creates complex tree structures and that can learn and adapt by changing their sizes, shapes and composition [38]. Different researchers have used GEP for modelling different properties of concrete incorporating various materials. For example, the study conducted by [39] used GEP for modelling the mechanical properties of green concrete incorporating waste foundry sand. Similarly, GEP has also been successfully used for modelling the resilient modulus of stabilized soils [40]. The GEP algorithm enabled the researchers to accurately predict the output (R^2^ > 0.85) and, at the same time, derive a new empirical equation for the output in terms of input variables.

In summary, GEP is a promising AI technique for the solution of engineering problems, yielding simple mathematical equations for future prediction. The RCP resistance of concrete was previously investigated using the black box AI model. Considering the black-box nature of different AI methods, this research study sought to model the RCP resistance of concrete specimens using different input variables such as age of the sample, amount of binder, fine aggregate and coarse aggregate, water-to-binder ratio, MK and the compressive strength value. In addition, GEP, being white-box model, was further used for deriving an empirical equation for RCP resistance of concrete in terms of the above-mentioned inputs. The developed GEP models were statistically evaluated, followed by a sensitivity and parametric analysis of the input variables.

## 2. Methodology

### 2.1. Database Compilation

**Figure 1:**
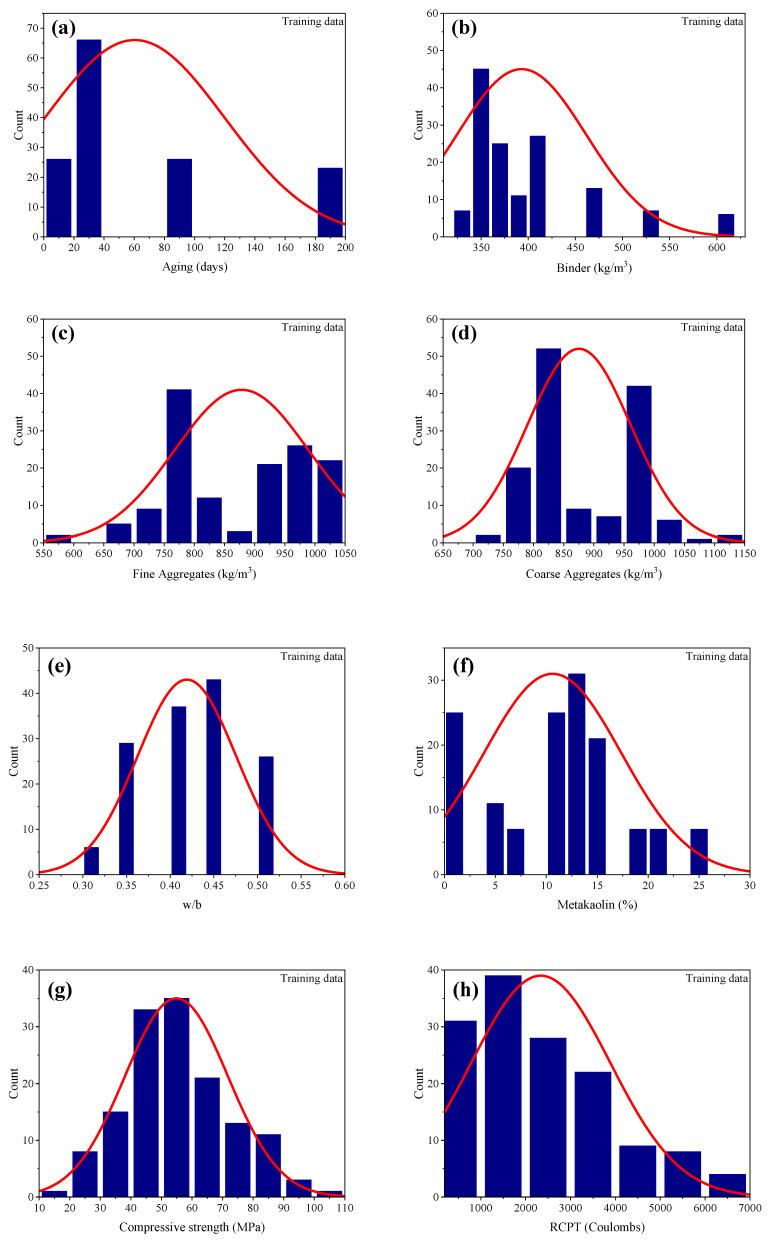

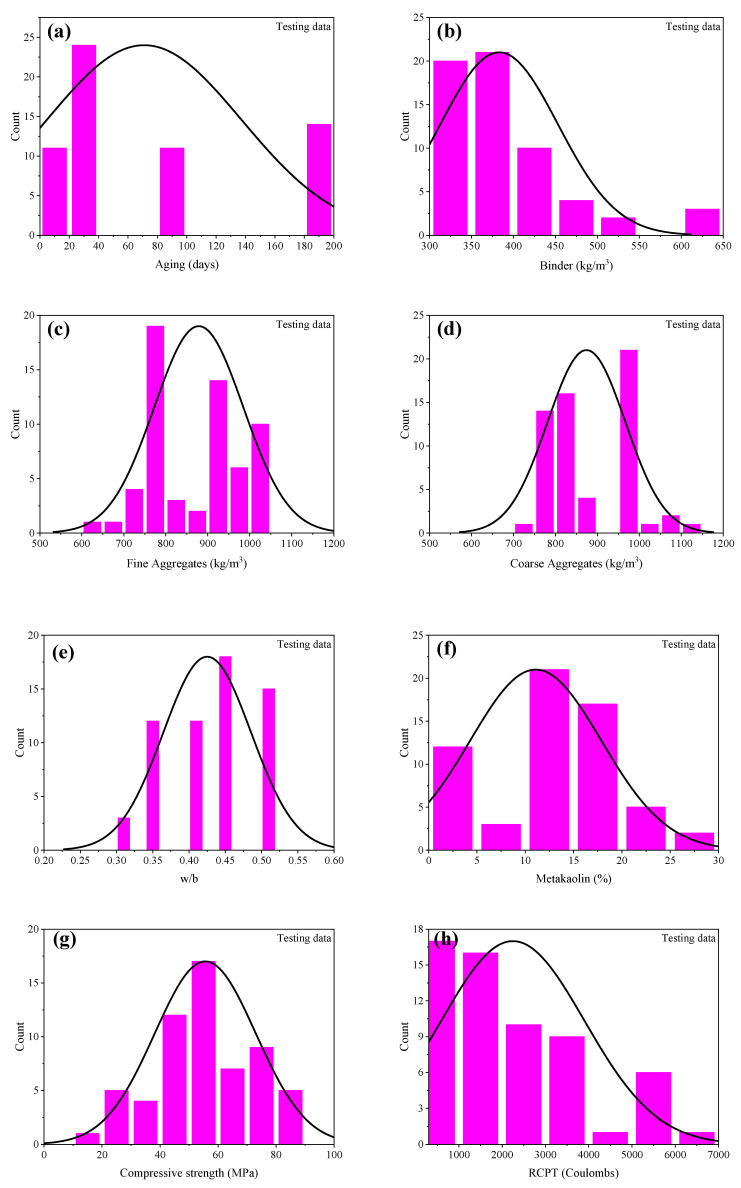
Frequency histograms of input and output variables; Training dataset (dark blue histograms) and test data (pink colored histograms) (**a**–**h**) for age, binder, fine aggregate, coarse aggregate, w/b ratio, metakaolin, compression strength and RCPT, respectively.

For an accurate model to precisely predict the output(s) based on a number of inputs, the database must be broad. The input variables must be evaluated statistically in order to determine their degree of influence on the output(s). In this study, the database was compiled from the experimental data of Al-Alaily and Hassan [41], Gilan et al. [42] and Ramezanianpour and Jovein [43]. The database comprised a total of 201 datapoints with seven different input variables and one output i.e., RCPT. The input variables included age of the sample (days), amount of binder (b), fine aggregate (Fag) and coarse aggregate (Cag) (Kg/m^3^), water-to-binder (w/b) ratio, MK (%) and the compressive strength value. Table 1 shows the descriptive statistics of the database that was used to develop the GEP models. Moreover, Figure 1 illustrates the frequency histograms of the input variables. It can be inferred from the histograms that except for the compressive strength, a majority of the input variables, such as age of the sample, b, Fag, Cag and w/b, were not normally distributed. Since the distribution of data depends upon the source used, it is not necessary that the data be normally distributed [23]. Similarly, it can be seen from Table 2 that the kurtosis value of binder was positive, while all other values of the input parameters were negative. Since, kurtosis represents the deviation of the distribution’s tail from the normal distribution tail, these values are also in accordance with the plots in Figure 1.

### 2.2. GEP Modelling

**Figure 2:**
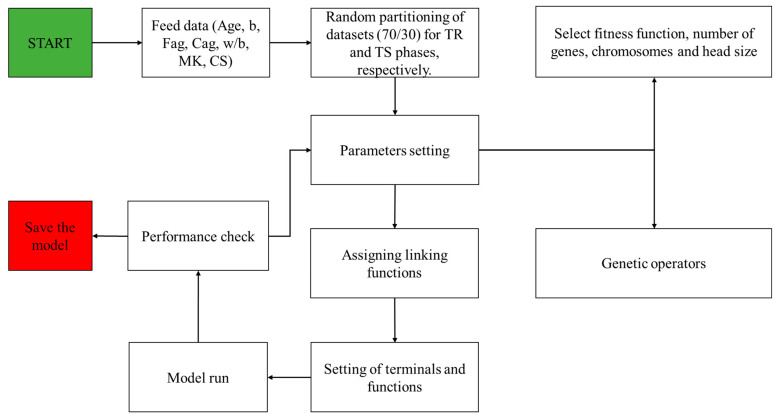
Flowchart of GEP modelling.

The GEP models were created with the help of GeneXprotools. Firstly, the data were fed into the GEP interface. The dataset was divided into two sets, namely the training (TR) dataset (70%) and the testing (TS) dataset (30%). As a result, 141 datapoints were used for the TR phase, whereas 60 datapoints were used for the TS phase. After that, the hyperparameter settings of GEP parameters was adjusted in order to formulate the most optimal model. For this reason, the numbers of chromosomes (N_c_) were varied from 30 to 200, numbers of genes (N_g_) from 3 to 5, and the H_s_ from 8 to 12. Different linking functions (+, −, ×, /) between the N_g_ were explored during the performance of trials, and it was found that the addition function provided the best performance. The flowchart of GEP modelling is shown in Figure 2, while the details of the undertaken trials are given in Table 2.

The parameters for GEP modelling were set using trial and error. As a result, the developed models overfit the data during the TR process and, subsequently, improved their performance in the TS phase. Gandomi and Roke [38] selected a model with a minimum objective function (OF) in order to address the problem of overfitting [39]. The OF varies from 0 to a maximum value, such that a model having OF ≈ 0 is deemed to yield the best performance. Different performance indices, such as coefficient of determination (R^2^), RMSE and mean absolute error (MAE), have been employed for evaluating the performance of the proposed GEP model. Table 3 shows the ideal values of these indices.

In the search for the best hyperparameters of the GEP model, a total of 11 trials (Models T1 to T11) were undertaken with varying N_c_, N_g_ and H_s_ (Table 2). Initially, the N_c_ were changed from 30 to 200 while keeping the H_s_ and N_g_ constant (i.e., 8 and 3, respectively). Similarly, the H_s_ was changed from 8 to 12, keeping the other two variables constant. A similar procedure was followed to find the optimal number of N_g_. Thus, the N_c_, H_s_ and N_g_ for an optimally performing GEP model (Model T3) were recorded to be 100, 8 and 3, respectively.

## 3. Results & Discussion

### 3.1. Effect of Variable Genetic Parameters

Table 2 depicts the influence of N_c_, H_s_, and N_g_ on the performance of the models (T1–T11) evaluated using different indices such as R^2^, RMSE and MAE, for both the TR and TS phases, respectively. The hyperparameter investigation was carried out in 11 distinct trials by varying the genetic parameters, i.e., N_c_, H_s_, and N_g_. The performance was observed in terms of the aforementioned statistical indices. It can be seen from Table 2 that initially the N_c_ was varied from 30 to 200, maintaining the other two genetic parameters constant (H_s_ = 8 and N_g_ = 3). Subsequently, the optimum model (Model T3) obtained during this investigation was further subjected to an increase in the H_s_ from 8 to 12. The N_g_ was changed from 3 to 5 in Models T9, T10, and T11 in order to obtain the optimal N_g_ value.

**Figure 3:**
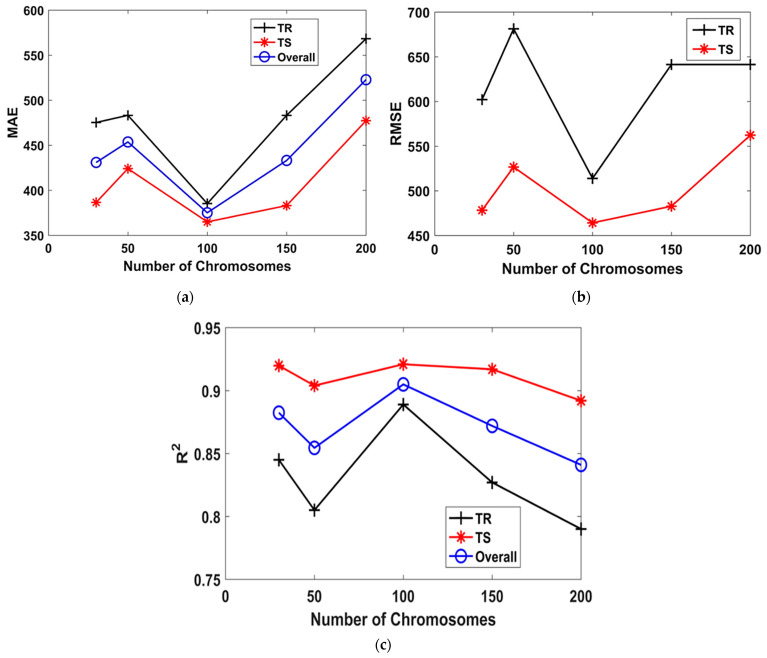
Effect of number of chromosomes on the performance of models. (**a**) MAE, (**b**) RMSE, (**c**) R^2^.

**Figure 4:**
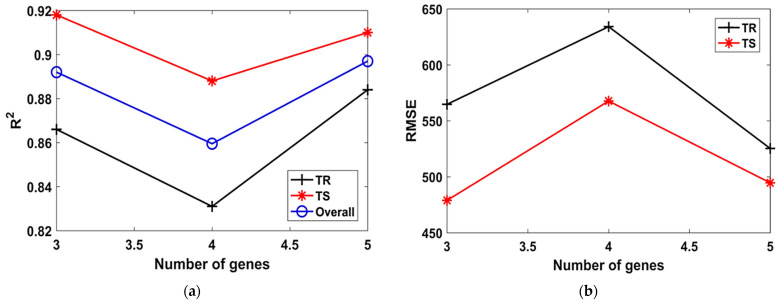

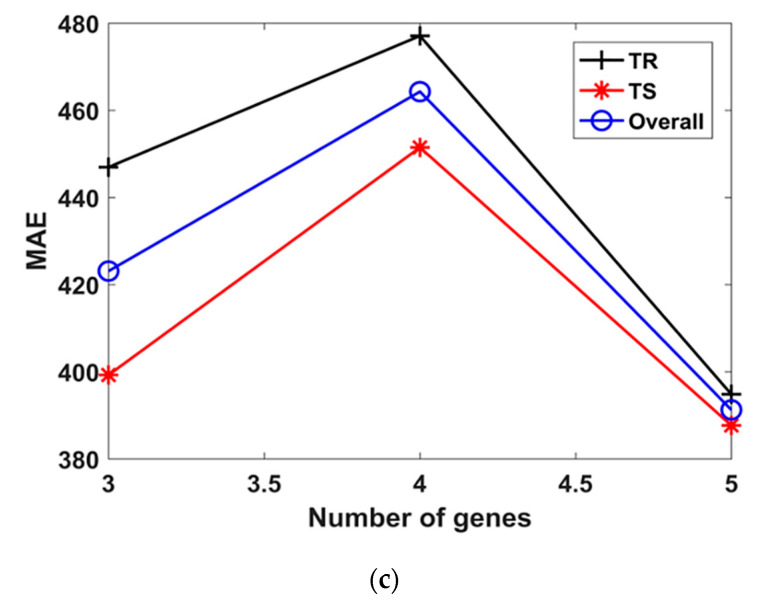
Effect of number of genes on the performance of models. (**a**) R^2^, (**b**) RMSE, (**c**) MAE.

**Figure 5:**
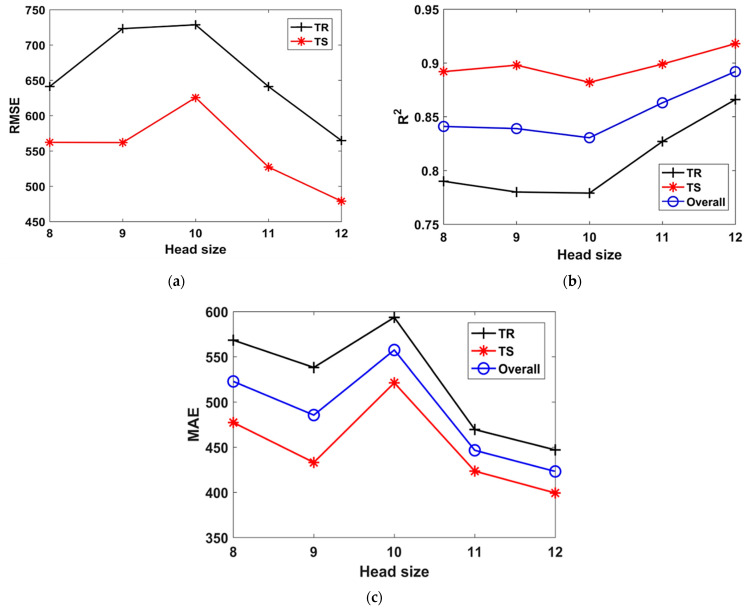
Effect of head size on the performance of models. (**a**) RMSE, (**b**) R^2^, (**c**) MAE.

Figure 3 depicts the performance in terms of MAE, RMSE, and R^2^ with changing N_c_ for TR phase, TS phase, and in case of the overall dataset. Figure 3 shows that when the N_c_ was increased from 30 to 200, the optimal N_c_ was revealed to be 100. Moreover, the detailed illustration of the N_c_ variation in Figure 3a–c shows that the overall values of MAE, RMSE, and R^2^ (i.e., 375.05, 489, and 0.905, respectively) indicated improved accuracy for N_c_ equaling 100. Afterwards, the performance of the other models plummeted. Similarly, the optimal values of H_s_ and N_g_ were observed as 8 and 3, as shown in Figure 4 and Figure 5, respectively. This strongly suggests that the hyperparameter tuning in the GEP modelling is solely a trial and error process, and there are no concrete recommendations regarding the effect of changing the genetic parameters. Furthermore, it is evident from the previous studies that increasing N_g_ and employing complex linking functions may increase the robustness of the models, but perplexes the traceable output mathematical equation [38,40].

Similarly, when the number of genes was increased from 3 to 5 in Model T9 to Model T11, and the number of chromosomes were kept constant at 100. It was found that the highest value of R^2^ = 0.88 in the TR phase and R^2^ = 0.92 in TS phase, as evident from Table 2. Lower values of R^2^ and higher values of RMSE and MAE were observed when the number of genes was 5. The effect of number of genes on the performance of the models can also be seen in Figure 4. It is evident that the models had lower R^2^ (Figure 4a) and higher RMSE (Figure 4b) when the number of genes was equal to 4.

Similarly, it can also be observed from Table 2 and Figure 5 that the value of H_s_ changed from 8 to 12 in Model T6 to Model T9. It can be inferred that when the H_s_ is increased while keeping the other two parameters constant, the R^2^ value increases for both the TR and TS phases (Figure 5b). The highest value of R^2^ = 0.87 and 0.92, can be observed for both TR and TS phase, respectively, in Model T9. Similarly, smaller values of RMSE = 564.8 and 478.9 and MAE = 447 and 399.3 were observed for both TR and TS phase, respectively, in Model T9. Looking at the performance of the model in different trials, it can be concluded from Table 2 that the trial T3 was the most optimal compared to the others since it had the highest R^2^ value and a smaller RMSE and MAE value.

### 3.2. Performance of Models

In order to evaluate the performance of the proposed models, a variety of statistical indices, such as the slope of regression line [39], statistical evaluation [44] and predicted to experimental (P/E) ratio [45], were employed.

#### 3.2.1. Statistical Evaluation

As discussed previously, Model T3 performed better (R^2^ = 0.89 for TR phase, and 0.92 for TS phase), followed by Model T11 (R^2^ = 0.88 for TR phase, and 0.91 for TS phase). The observed values of R^2^ indicate a good agreement between the predicted and actual values. However, deciding about the performance of a model based on “R^2^” alone is not sufficient, and other statistical error indices must also be considered. In this regard, the values of RMSE and MAE were studied to evaluate the performance of the different models, in addition to the R^2^ value (Table 2). It is evident from Table 2 that besides having a higher R^2^ value, Model T3 exhibited the lowest RMSE (513.9 in TR phase, and 464.1 in TS phase) and MAE (385.2 in TR phase and 364.9 in TS phase). Similarly, Model T11 performed as the second-best model with R^2^ = 0.88, RMSE = 525.3 and MAE = 394.8 in the TR phase, while Model T1 performed as second-best model with R^2^ = 0.92, RMSE = 478.2, and MAE = 386.6 in the TS phase, respectively. The ranking of the models based on the different statistical indices is shown in Table 4.

#### 3.2.2. Comparison of Regression Slopes

AI models can be assessed using the slope of the line trending between the actual and forecasted values. A similar assessment method was employed for this study, and regression slopes were plotted for all 11 trials. It is pertinent to mention that, an ideal line having slope value of unity can be shown by plotting a line at an angle of 45 degrees with the *x*-axis. The performance of the model is expected to be superior, i.e., the predicted values will be closer to the actual values if the plotted points are close to the standard line. A regression line whose slope approaches the value 1 and its correlation value (i.e., R) ≥0.8 are considered reliable in predicting new data [46,47,48].

**Figure 6:**
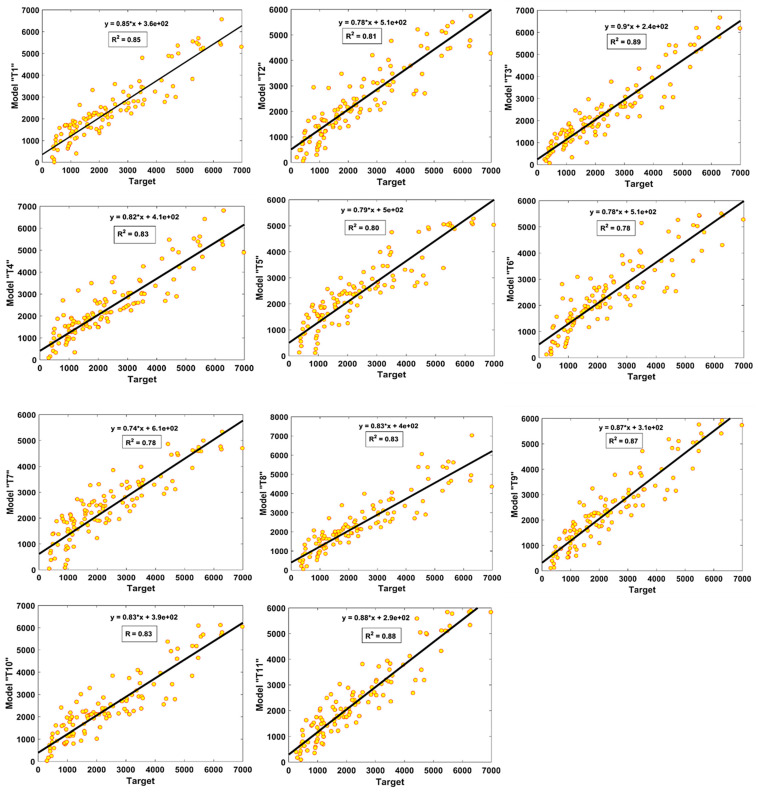
Comparison of regression slopes for the developed models in TR phase.

**Figure 7:**
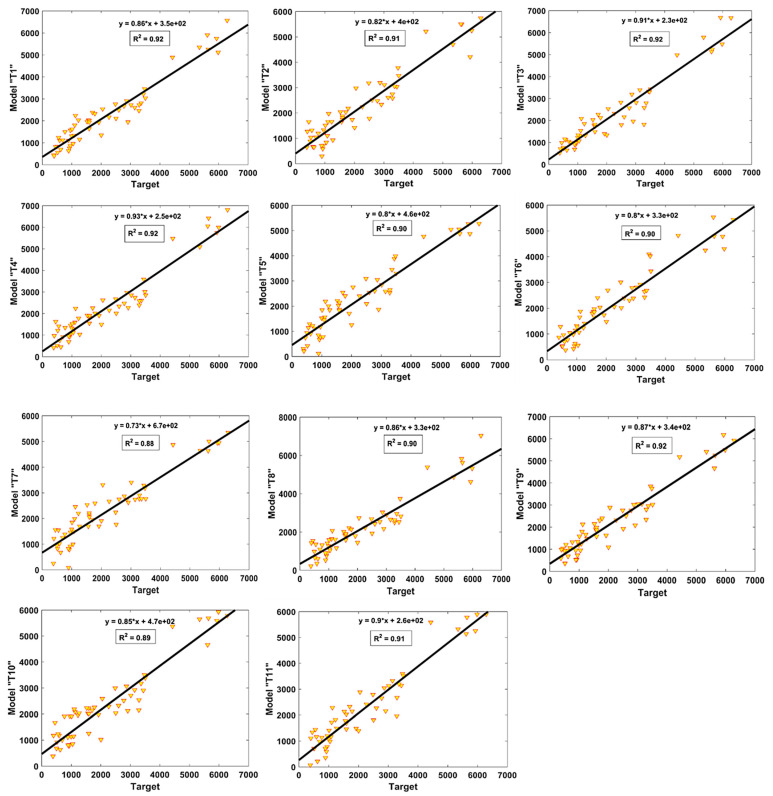
Comparison of regression slopes for the developed models in TS phase.

Figure 6 and Figure 7 show the value of R^2^ and regression slopes for both the TR and TS phases, respectively. It is evident that the value of R^2^ exceeded 0.8 for most of the models. In the TR phase, Model T3 had the finest fit with an R^2^ value of 0.89, whereas Model T6 and Model T7 had lower values of R^2^ = 0.78, each. Similarly, the performance of the models seemed to be better in the TS phase, as evident from their higher R^2^ values, and all the models had R^2^ ≥ 0.85. Similarly, it can also be observed from Figure 6 and Figure 7 that the highest values of slope “m” = 0.89 and 0.92 were obtained for the optimal model “T3” in both the TR and TS phases, respectively. It is important to mention here that for m = 1, the slope of the regression line will be exactly 45^°^. The values of “m” observed for the model “T3” were closer to one as compared to other models; therefore, it can be concluded from the higher R^2^ and m values that this was the optimal performing model compared to the others.

#### 3.2.3. Model Predicted to Experimental (P/E) Ratio

**Figure 8:**
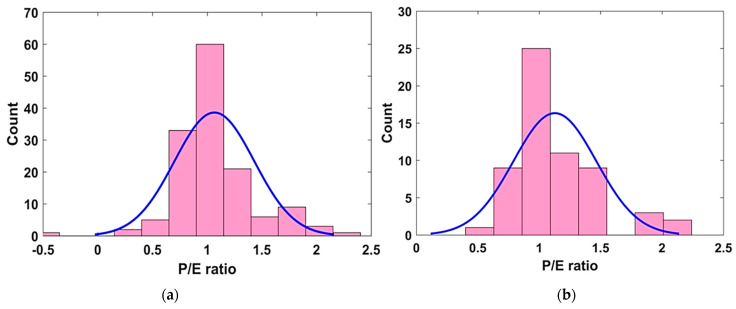
P/E ratio distribution of best performing model T3. (**a**) TR phase, (**b**) TS phase.

The performance of the different models formulated during different trials was further investigated using the P/E ratio. Figure 8 shows the distribution of the P/E ratio for the best performing Model T3. The bin range was kept as 0, 0.5, 1.0, 1.5, 2.0, and 2.5. It can be seen that most of the P/E values in predicting RCP values were concentrated mainly in the bin range proximal to one, in both the TR and TS phases. This aided another statistical check in evaluating the performance of the model and served as visual confirmation of the best performance model, i.e., Model T3.

#### 3.2.4. Visual Interpretation of Results via Taylor Diagram

**Figure 9:**
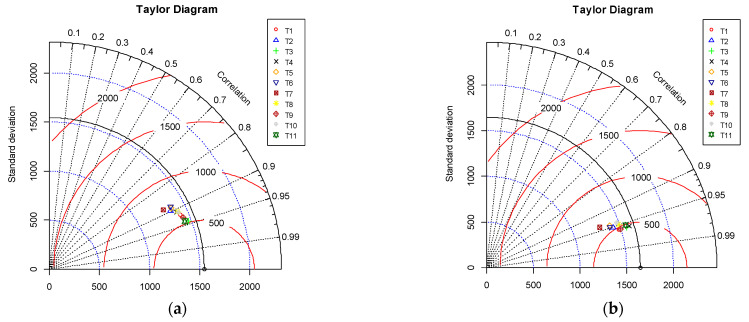
Taylor diagram for the proposed models. (**a**) Training phase, (**b**) Testing phase.

To better visualize the results of the 11 proposed models, a Taylor diagram was employed. It is a two-dimensional graph providing a concise overview of a model’s accuracy. It represents the relationship between the actual and forecasted values using the correlation coefficient, RMSE and the standard deviation ratio. For performance comparison, the developed model’s value is marked on the diagram, and its performance is compared against the reference/benchmark point, which has already been plotted on the diagram. Figure 9 shows the Taylor diagram of the developed models for both TR and TS phases, respectively. It is evident that model T3 had the highest correlation coefficient value and a lower RMSE, as shown in Figure 9a, followed by model T11, whose standard deviation value was second closest to the reference value, as shown in Figure 9a. This visual interpretation of the model’s performance from the Taylor diagram supported the ranking of the proposed models, as shown in Table 4. Similarly, in the TS phase, model T3’s correlation coefficient value was the highest among all models, as shown in Figure 9b; however, the standard deviation of model T4 was closer to the reference value compared to model T3.

### 3.3. GEP Formulations

In order to obtain an empirical equation for predicting the RCP of concrete using the different input variables considered in this study, the best performing model, i.e., Model T3, was used for generating the empirical equation. For this purpose, the expression tree for Model T3 (Appendix A) and the MATLAB model were utilized to obtain the mathematical expression that could be further used for forecasting the RCP values and performing the sensitivity as well as parametric analysis. As a result, we derived Equation (1), which can predict the RCP values of concrete specimens using various input variables (i.e., age, amount of binder, Fag, Cag, w/b, MK and compressive strength). It is highly recommended to use the prediction equation for input variables whose ranges and mechanical properties are discussed in Section 2.1 (Database compilation) [48,49].

(1) $$\begin{array}{l} RCPT=(Fag+A)+((Cag-T)*(w/b))-B+MK^{2}+Fag-370.95-C\\ Where,A=((w/b)/T{)}^{2}*(Cag+b)*(Cag-b),b=(13.68*MK)*(w/b{)}^{2}*40.9,C=Cag+(6.18*CS) \end{array}$$

### 3.4. Parametric and Sensitivity Analyses

Parametric analysis is generally performed to verify the reliability of various AI models. Parametric analysis of all of the input parameters (age, b, Fag, Cag, w/b, MK and compressive strength) was carried out in order to assess their influence on the resulting RCPT of concrete specimens. Table 5 shows the possible combinations of the different input features that were adopted for the parametric analysis. For this purpose, the simulated dataset was generated such that one of the input variables (first variable, age) was varied between its extreme values in equal increments while keeping the remaining variables at their average values. In the next step, the amount of binder (second variable, and so on) was changed in a similar manner. This procedure was performed for all of the input variables. The corresponding change in the target variable was calculated using the prediction equation (Equation (1)). The changing input variables and the corresponding variation in the RCP values were plotted to obtain the parametric analysis. The net change in the target variable due to changing a particular input attribute was calculated in terms of weighted percentage in order to obtain the sensitivity of each variable. Note that the sensitivity analysis showed the response of the prediction model by varying the input features [40,50].

**Figure 10:**
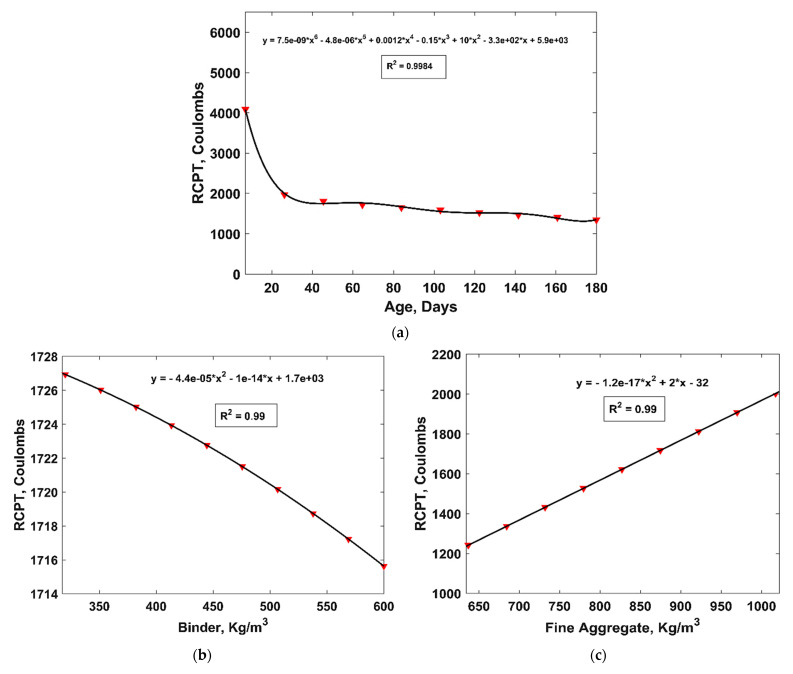

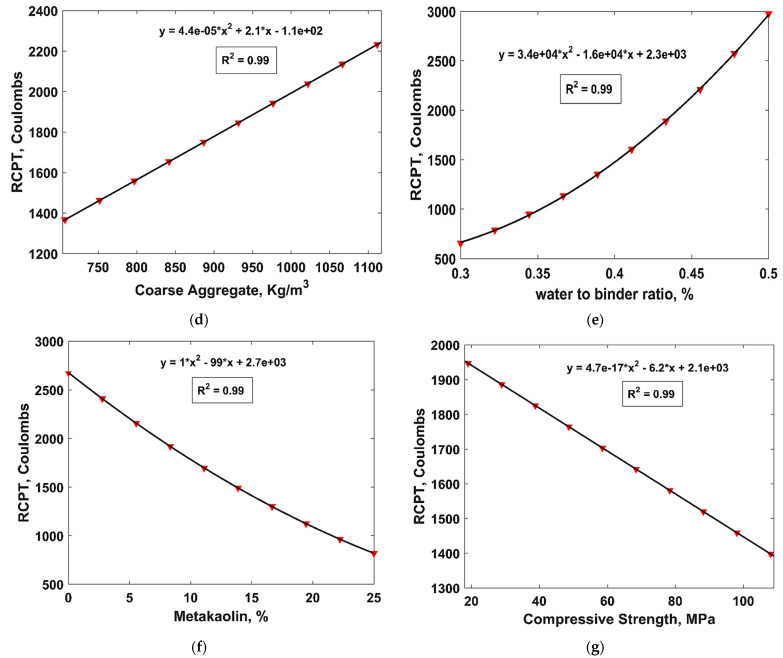
Parametric analysis of input variables. (**a**) age, (**b**) binder, (**c**) Fag, (**d**) Cag, (**e**) w/b ratio, (**f**) MK, and (**g**) compressive strength.

**Figure 11:**
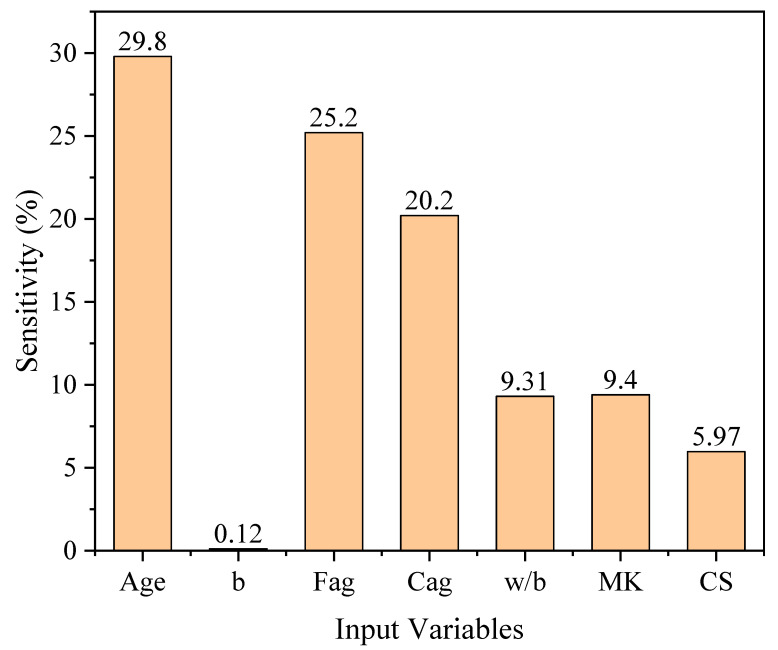
Sensitivity analysis of all input variables.

Figure 10 shows the variation in the RCP values in response to the change in each input variable. It is evident from Figure 10 that the RCP value decreased with the curing duration and increasing amount of b, compressive strength, and MK, while it increased with the amount of Fag, Cag and w/b ratio. The parametric analysis of the age of sample showed that the resistance of concrete specimens to RCP increases rapidly within the first 28 days after casting; however, it remains almost the same after 120 days. Similarly, polynomial equations were fitted to the resulting parametric analysis, which depicted good agreement with the datapoints, i.e., all of the cases had R^2^ above 0.97. Figure 11 shows the sensitivity of each variable in resisting chloride penetration. Concrete age is the most significant parameter that influences RCP values, followed by Fag, Cag, w/b ratio, MK content, and finally, the compressive strength of concrete.

## 4. Conclusions

This study was undertaken to evaluate the influence of different variables, such as age of the sample (age), amount of binder (b), fine aggregate (Fag) and coarse aggregate (Cag), water to binder ratio (w/b), metakaolin (MK), and the compressive strength on the RCP resistance of concrete using gene expression programming (GEP). For this purpose, the number of chromosomes (N_c_), head size (H_s_), and number of genes (N_g_) of the GEP model were varied to study their influence on the predicted values of the RCP. Following are the main conclusions drawn from this study:

1. The tuning of the hyperparameter settings for the GEP model revealed that the model with N_c_ = 100, H_s_ = 8 and N_g_ = 3 (Model T3) resulted in an optimal GEP model, as evident from its high R^2^ values (i.e., 0.89 in the TR phase and 0.92 in the TS phase, respectively). Similarly, the values of RMSE = 513.9 and 464.1, and of MAE = 385.2 and 364.9, were also comparatively smaller than in all the other models in the TR and TS phases, respectively.
2. The regression slope analysis showed that the predicted values were in good agreement with the experimental values, as indicated from the higher R^2^ values. It was also observed that the performance of the models improved in the TS phase, which was reflected in their higher R^2^ values, with the majority of developed models having R^2^ > 0.8. In addition, the P/E ratio analysis revealed that Model T3 was the best performing model, because a larger frequency was observed for the P/E ratio proximal to one.
3. Similarly, the parametric analysis for the best performing Model T3 revealed that the amount of binder, compressive strength and age of the sample enhanced the RCP resistance of concrete specimens. However, among the different input variables, the RCP resistance sharply increased within the first 28 days of age of the concrete specimen.

## Figures and Tables

**Table 1:** Descriptive statistics of the input variables.

Descriptive Statistics	Age	b	Fag	Cag	w/b	MK	Compressive Strength	RCPT
Average	63.53	389.92	0.42	10.76	878.46	874.97	55.11	2309.98
Standard Error	4.35	4.88	0.004	0.48	7.70	6.18	1.19	111.36
Median	28	360	0.45	12.5	881.30	832.5	52.7	1973
Standard Deviation	61.63	69.21	0.058	6.74	109.21	87.69	17.01	1578.79
Sample Variance	3798.46	4790.43	0.003	45.41	11,925.88	7689.79	289.30	2492.57 × 10^3^
Kurtosis	−0.42	2.19	−0.90	−0.49	−0.99	−0.82	−0.31	0.11
Skewness	1.04	1.69	−0.24	−0.10	−0.25	0.34	0.25	0.93
Minimum	7	320	0.3	0	589.2	707	19	203
Maximum	180	600	0.5	25	1017.5	1111.7	108	6982

**Table 2:** Details of 11 GEP trials conducted for evaluating the most optimal model.

Trial/Model	No. of Variables	No. of Chromosomes	Head Size	No. of Genes	TR Phase			TS Phase		
					R^2^	RMSE	MAE	R^2^	RMSE	MAE
T1	7	30	8	3	0.84	602.1	475.3	0.92	478.2	386.6
T2	6	50	8	3	0.81	681.3	483.3	0.90	526.6	424
T3	7	100	8	3	0.89	513.9	385.2	0.92	464.1	364.9
T4	7	150	8	3	0.83	641.3	483.3	0.92	482.7	383.1
T5	6	200	8	3	0.79	641.3	568.3	0.89	562.2	477.3
T6	7	100	9	3	0.78	723.3	538.0	0.89	561.9	433.1
T7	7	100	10	3	0.78	728.7	593.5	0.88	625.6	521.3
T8	7	100	11	3	0.83	641.2	469.6	0.89	527.2	423.5
T9	7	100	12	3	0.87	564.8	447	0.92	478.9	399.3
T10	6	100	8	4	0.83	634.2	477.1	0.89	567.7	451.5
T11	7	100	8	5	0.88	525.3	394.8	0.91	494.6	387.7

**Table 3:** Ideal values of performance indices.

Index	Range/Ideal Value
R^2^	(0–1)/1
RMSE	(0–∞)/0
MAE	(0–∞)/0

**Table 4:** Ranking of the GEP models (for 11 trials) based on R^2^ and RMSE.

Statistic	R^2^		RMSE		MAE	
Rank	1st	2nd	1st	2nd	1st	2nd
TR Phase	T3	T11	T3	T11	T3	T11
TS Phase	T3, T1	-	T3	T1	T3	T4

**Table 5:** Dataset used for parametric analysis.

Input Variables		Constant Input Parameters	No. of Datapoints
Parameter	Range		
Age	7–180	B = 389.93, w/b = 0.42, MK = 10.76, Fag = 878.46, Cag = 874.97, compressive strength = 55.12	9
b	320–600	Age = 63.53, w/b = 0.42, MK = 10.76, Fag = 878.46, Cag = 874.97, compressive strength = 55.12	
Fag	589.2–1017.5	Age = 63.53, B = 389.93, w/b = 0.42, MK = 10.76, Cag = 874.97, compressive strength = 55.12	
Cag	707–1111.7	Age = 63.53, B = 389.93, w/b = 0.42, MK = 10.76, Fag = 878.46, compressive strength = 55.12	
w/b	0.3–0.5	Age = 63.53, B = 389.93, MK = 10.76, Fag = 878.46, Cag = 874.97, compressive strength = 55.12	
MK	0–25	Age = 63.53, B = 389.93, w/b = 0.42, Fag = 878.46, Cag = 874.97, compressive strength = 55.12	
compressive strength	19–108	Age = 63.53, B = 389.93, w/b = 0.42, MK = 10.76, Fag = 878.46, Cag = 874.97	

## Data Availability

The data used in this research have been properly cited and reported in the main text.

## Abbreviations

Rapid chloride ion penetration	RCP
Number of chromosomes	N_c_
Number of genes	N_g_
Head size	H_s_
Gene expression programming	GEP
Amount of binder	b
Fine aggregate	Fag
Coarse aggregate	Cag
Water to binder ratio	w/b
Metakaolin	MK
Model predicted to experimental	P/E

## Appendix A

Expression Trees Derived for GEP Modelling Used for the Development of Mathematical Equation are Given Below.
